# A kaleidoscopic view of male urethral pathologies on 64-slice multidetector computed tomographic urethrography: A novel technique

**DOI:** 10.4102/sajr.v25i1.1964

**Published:** 2021-01-29

**Authors:** Shuchi Bhatt, Avinaba Banerjee, Anupama Tandon, Saumya Dangwal, Arun Gupta

**Affiliations:** 1 Department of Radiology, University College of Medical Sciences and GTB Hospital, Delhi, India; 2 Department of Surgery, University College of Medical Sciences and GTB Hospital, Delhi, India

**Keywords:** male urethra, urethrography, computed tomography, stricture, 3D imaging

## Abstract

Pathologies of the male urethra are mostly obstructive in nature and require imaging to delineate the lesion type, site, extent and associated abnormality of the urinary bladder. Contrast urethrography (CU) is the gold standard investigation for urethral assessment but has many limitations. Cross-sectional imaging is infrequently used for the evaluation of the urethra but has been gaining importance recently. Multidetector computed tomographic urethrography (MDCTU) has the capability of evaluating the entire length of a male urethra in a single setting and overcomes many technical and patient limitations of CU. Being a novel technique, most radiologists are not familiar with MDCTU and the imaging spectrum of various urethral and bladder pathologies. This pictorial review attempts to present the imaging appearance of the normal male urethra and spectrum of pathological findings, with highlights on its advantages over the CU technique.

## Background

Contrast urethrography (CU) – retrograde urethrography and micturating cystourethrography – evaluates the anterior and posterior male urethra, respectively, and is the gold standard for urethral assessment.^1^ Cross-sectional techniques – sonourethrography, transrectal voiding sonourethrography and magnetic resonance urethrography – delineate the urethral pathologies but have inherent limitations. Sonourethrography has a small field of view, assesses only the anterior urethra and is operator dependent.^2^ Transrectal voiding sonourethrography^3^ evaluates the posterior urethra with much patient discomfort. Magnetic resonance imaging is not frequently used because of its cost and complexity.

Multidetector computed tomographic urethrography (MDCTU) is obtained using a multidetector computed tomographic scanner to acquire fast, thin slices of a distended urethra as the patient voids^4^ a contrast-filled urinary bladder. Better sensitivity to the contrast and post-processing of iso-volumetric axial images to obtain reformatted two-dimensional (2D) and 3D images explicitly demonstrate a plethora of urethral pathologies. Comprehensive evaluation of the entire length of the urethral lumen, periurethral, pelvic soft tissues and bones is possible with MDCTU.^4^

The possibility of post-processing in MDCTU allows the patient to micturate in a comfortable position with representation of non-distorted urethral anatomy, leading to better patient compliance. Therefore, MDCTU proves useful in uncooperative patients or in the following clinical situations:

- when procedural failure is expected because of technical difficulties: unsuccessful catheterisation, inadequate bladder filling and inability to assume an oblique position during micturition because of a pelvic fracture
- following suboptimal examination on CU resulting in inadequate urethral evaluation: failure to delineate the pathology, especially of the posterior urethra because of obscuration from the pelvic bones; faint contrast density in the urethra and contrast extravasation into periurethral tissues, hindering urethral visualisation
- if surgical intervention is planned: improved surgical planning can be done in cases such as pelvic trauma with suspected posterior urethral distraction defects; accurate measurement of the stricture length ensures optimal treatment decisions, especially when associated periurethral diseases such as fistula and false passages are present.

## Technique

Initially, bladder filling is achieved either directly by instillation of contrast (250 millilitres [mL] – 400 mL of 10% diluted ionic or non-ionic contrast media) through a supra-pubic catheter or a Foley’s catheter or indirectly by intravenous contrast administration. Thin axial computed tomography (CT) sections (e.g. 64 × 0.6 with a 64-slice CT) are obtained as the patient voids into a condom catheter in a comfortable position (supine, prone, oblique or lateral).

Multiplanar reformatting (MPR), maximum intensity projection (MIP) and volume rendering technique (VRT) images are used for vivid representation of the urethra and its pathology. Multiplanar reformatting images depict the urethra and periurethral soft tissues, enabling visualisation of normal anatomy and pathology simultaneously. Curved MPR images show the entire length of the curved urethra and thus delineates a stricture, if present. Thick MIP reconstructions clearly depict the capacity, outline of the contrast-filled bladder, vesico-ureteric reflux into the lower ureters as well as the urethral outline and calibre. The volume rendering technique is used to provide a 3D external view of the bladder and urethra. Bone removal algorithms help in the optimal visualisation of the posterior urethra.

A 64-slice multidetector computed tomography scanner, Somatom Definition AS (M/s Siemens AG Healthcare Sector, Germany), was used to acquire all the scans presented in this review. The various types of images present exquisite details of the common urethral pathologies encountered in a male patient.

## Normal imaging appearance

**FIGURE 1:**
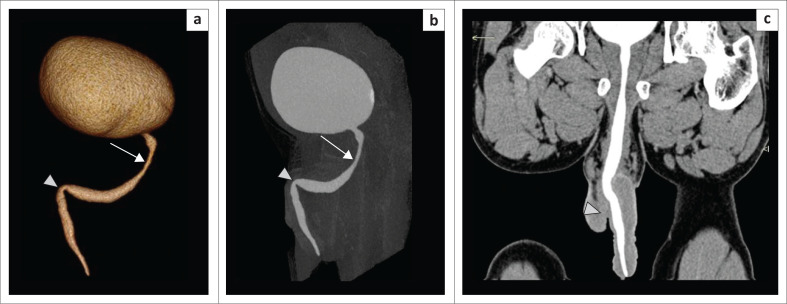
Multidetector computed tomographic urethrography (MDCTU) of a normal urethra: (a) volume rendering technique image in the sagittal view, (b) maximum intensity projection image in the sagittal view and (c) curved multiplanar reformatted (MPR) image. The urethra is seen arising from the bladder neck as a contrast-filled tubular structure and opening into the external urethral meatus. The membranous urethra is the narrowest part with near-parallel walls, at the level of the external urethral sphincter. The bulbar urethra is the widest portion having a normal coned appearance at the bulbomembranous junction (arrow). The bend or kink at the peno-scrotal junction (arrowhead) is normal since the penis is not stretched on MDCTU. The curved MPR image (c) confirms the kink as normal calibre.

The urethra can be visualised on VRT (Figure 1a), MIP (Figure 1b) and curved MPR (Figure 1c) images as a contrast-filled tubular structure, arising from the neck of the bladder and opening into the external urethral meatus. The bulbar urethra is the widest portion, having a normal coned appearance at the bulbomembranous junction. The membranous urethra is the narrowest part with near-parallel walls, at the level of the external urethral sphincter (Figures 1a, b and c). Distally, the bulbar urethra continues as the penile urethra with a normal ‘kink’ or ‘bend’ at the penoscrotal junction, which should not be confused with a stricture (Figures 1a, b and c).

The VRT and MIP images can be represented with bone subtraction for depicting a clear 3D volumetric view (VRT) of the urethra, whilst the MIP image shows the contrast-filled urethra and bladder as a composite CU image without the overlapping pelvic bones. This provides a clear understanding of any abnormal morphology of the urethra.

## Urethral pathologies

### Stricture

**FIGURE 2:**
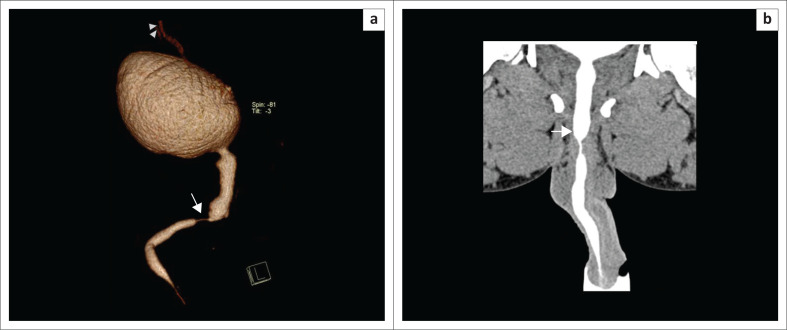
(a) Volume rendering technique and (b) curved multiplanar reformatted image. Images demonstrate a tight penobulbar stricture (arrow), with a dilated posterior urethra. The opacified distal ureters (arrowheads) are visible as the patient was administered intravenous contrast, and they should not be interpreted as vesico-ureteric reflux (VUR). This patient could not be catheterised at retrograde urethrography because of the tight stricture.

**FIGURE 3:**
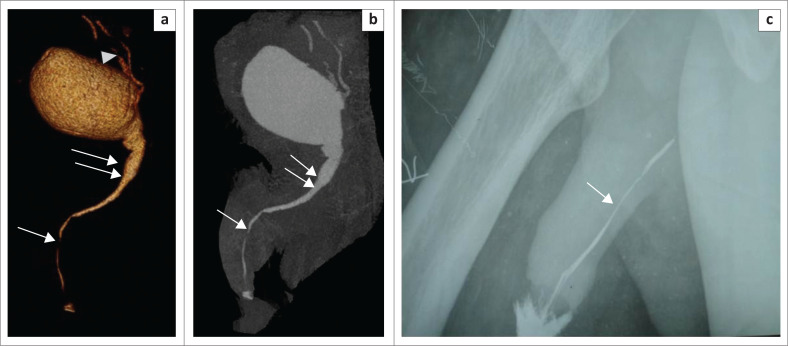
(a) Volume rendering technique image (multidetector computed tomographic urethrography [MDCTU]) showing a long-segment anterior urethral stricture (arrow) with adequate visualisation of the urinary bladder and posterior urethra (double arrows); both lower ureters (arrowheads) are opacified because of intravenous contrast administration. (b) Sagittal maximum intensity projection (MDCTU) showing the long-segment anterior urethral stricture (arrow) with adequate visualisation of the urinary bladder and posterior urethra (double arrows). (c) Retrograde urethrography image of the same patient indicating the long-segment anterior urethral stricture (arrow), with no passage of contrast into posterior urethra. Micturating cystourethrography could not be performed in this patient due to unsuccessful catheterisation.

**FIGURE 4:**
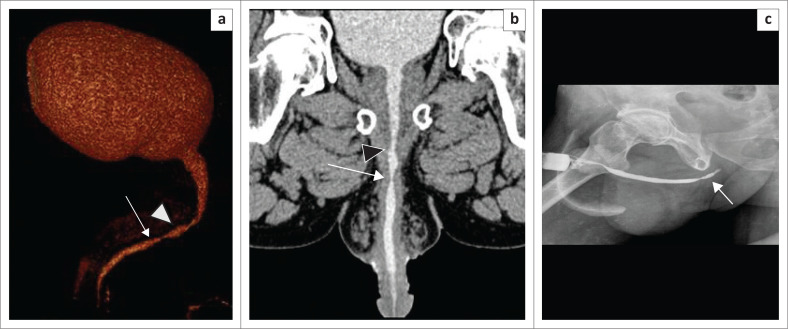
(a) Volume rendering technique image (multidetector computed tomographic urethrography [MDCTU]) showing two strictures, one at the penobulbar junction (arrow) and the other in the bulbar urethra (arrowhead). (b) Curved multiplanar reformatted image (MDCTU) of the same patient showing the two strictures. (c) Retrograde urethrography image of the same patient demonstrating the stricture at the penobulbar junction (arrow), with no distal passage of contrast into the posterior urethra.

**FIGURE 5:**
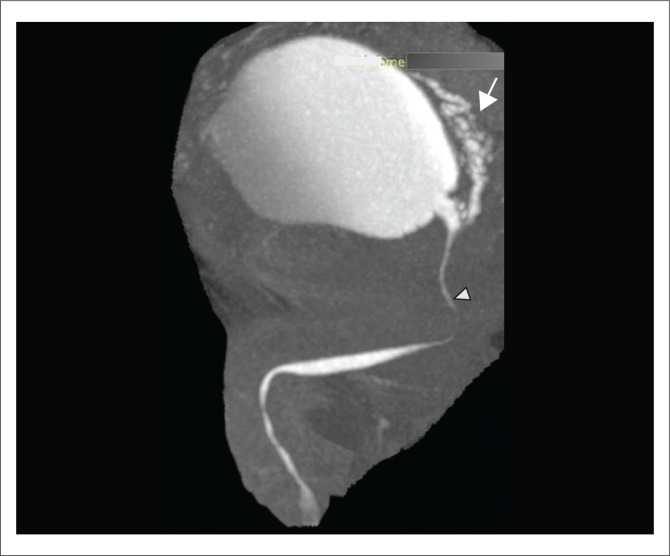
Sagittal maximum intensity projection (multidetector computed tomographic urethrography) showing reflux into the prostatic glands and seminal vesicles (arrow) and a urethral stricture of the entire posterior urethra. (arrowhead).

A urethral stricture is the commonest obstructive lesion, appearing as narrowing or abrupt change in calibre of the contrast-filled urethral lumen. A short- or long-segment stricture is less than or more than 2 cm, respectively^1^ (Figures 2 and 3). The definitive treatment is urethroplasty, and surgical planning requires accurate demonstration of the stricture length.^5,6,7^ The mean stricture length can be more accurately measured on MDCTU than on CU.^1^ Greater sensitivity for contrast in an antegrade urethrographic study on MDCTU^1,4^ allows for posterior urethral evaluation as well, unlike in micturating cystourethrography. Partial urethral strictures (Figures 2–4) and multiple urethral strictures (Figure 4) can be delineated on MDCTU. A posterior urethral stricture, its length, extent and the associated reflux of contrast into the prostatic glands and seminal vesicles is also well demonstrated on MDCTU (Figure 5).

### Urethritis

**FIGURE 6:**
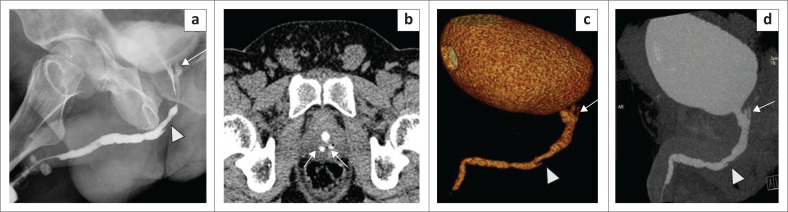
(a) Retrograde urethrography (RGU) image (right lateral view) showed an outpouching from the prostatic urethra (arrow), likely a urethral diverticulum. Anterior urethritis and a false tract (arrowhead) in the bulbar urethra were also noted. (b) Axial section (multidetector computed tomographic urethrography [MDCTU]) of the same patient showed reflux into both ejaculatory ducts (arrows), which mimicked the appearance of urethral diverticulum on RGU. (c, d) Volume rendered technique (VRT) and maximum intensity projection (MIP) images, respectively (MDCTU), of the same patient also showed reflux into bilateral ejaculatory ducts (arrows), which mimicked the appearance of a urethral diverticulum on RGU. Anterior urethritis and a false tract in the bulbar urethra (arrowhead) were also noted on VRT and MIP.

**FIGURE 7:**
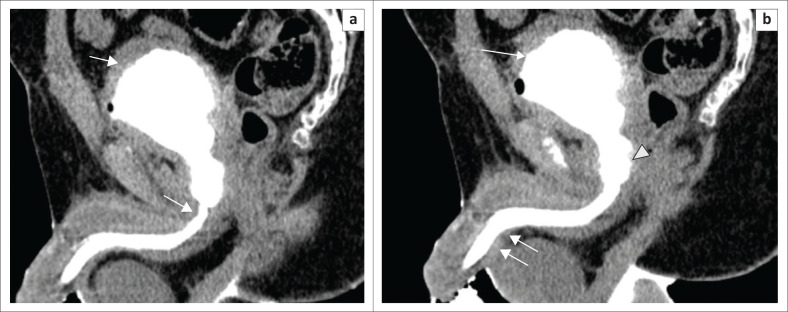
Sagittal multiplanar reformatted images: (a) Partial stricture (short arrow) at the bulbomembranous junction, with a dilated proximal urethra. Small-sized bladder with cystitis (arrow) and posterior urethritic changes (irregularities in urethral wall) (arrowhead) are noted. (b) Extravasation into the periurethral soft tissues noted at the level of mid-penile urethra (double arrows).

Urethral inflammation on MDCTU is seen as irregularity in the urethral wall (Figure 6). It is often associated with contrast extravasation into the periurethral soft tissues or Littre’s glands. Cystitis is commonly seen with urethritis and urethral strictures (Figure 7).

### False tract

**FIGURE 8:**
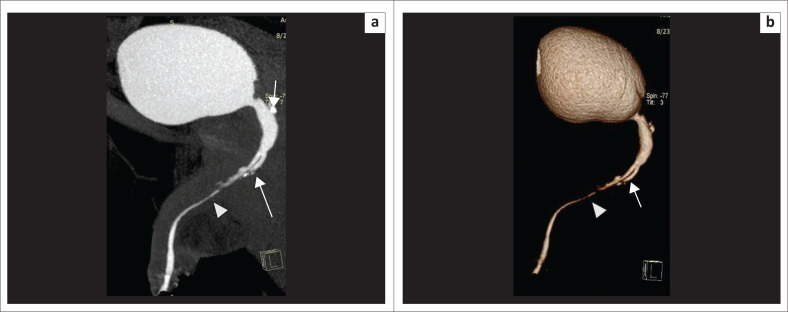
(a) Sagittal maximum intensity projection image showing a partial penobulbar stricture (arrowhead), anterior urethritis, with a false tract in the bulbar urethra (long arrow), cystitis and prostatic calcification (short arrow). (b) Volume rendered technique image showing partial penobulbar stricture (arrowhead) and anterior urethritis, with a false tract in the bulbar urethra (long arrow).

A false tract may result from an attempt to dilate the urethral stricture. False tracts are seen as contrast-filled linear tracts, separate from the main urethra but communicating with the main urethral lumen (Figures 6 and 8). Multidetector computed tomographic urethrography accurately helps to differentiate it from reflux into any normal anatomical structures (Figure 6).

### Posterior urethral distraction defects

**FIGURE 9:**
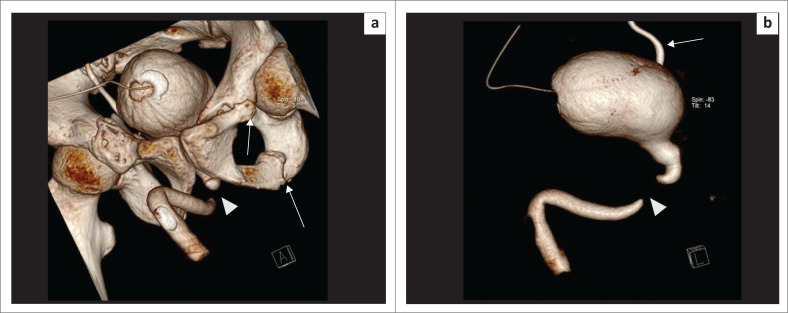
Volume rendered technique image (a) before bone removal and (b) after bone removal, showing the posterior urethral distraction defect (arrowhead) at the level of the membranous urethra. The part of the urethra proximal to the defect is dilated. Right-sided vesico-ureteric reflux (VUR) (arrow) and the fractures of bilateral superior pubic rami and left inferior pubic rami (arrows) are noted.

**FIGURE 10:**
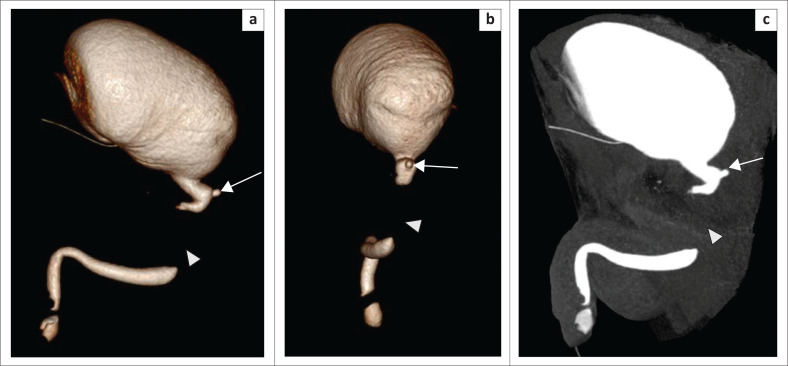
(a, b) Volume rendered technique images (multidetector computed tomographic urethrography) showing a posterior urethral distraction defect (arrowhead) and a urethral diverticulum (arrow) in different planes. (c) Maximum intensity projection image of the same patient showing the posterior urethral distraction defect (arrowhead) and urethral diverticulum (arrow) clearly.

These defects are difficult to evaluate on CU as associated pelvic fractures result in suboptimal radiographs.^8^ Soft tissues are well demonstrated by selective removal of overlapping bones on MDCTU, thus enhancing visualisation of the posterior urethra. Posterior urethral distraction defects are best seen on 3D-VRT images^8^ (Figures 9 and 10).

### Urethral diverticulum

A urethral diverticulum is uncommon and classified as congenital or acquired or as primary or secondary.^9^ Recurrent infections, instrumentation or surgery, inflammation and trauma of the periurethral glands or ducts result in secondary diverticula formation.^10^ It appears as a focal contrast-filled outpouching from the urethra (Figure 10) and is continuous with the urethral lumen. Associated complications such as calculi are also well demonstrated on MDCTU.

### Urethral calculi

**FIGURE 11:**
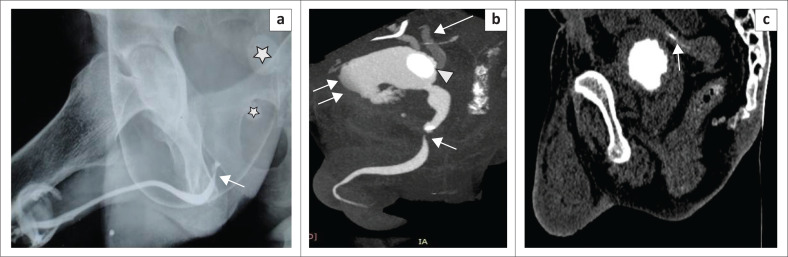
(a) Retrograde urethrography (RGU) image showing a normal anterior urethra with tapering at the bulbomembranous junction (arrow) and no distal passage of contrast into the posterior urethra. Micturating cystourethrography could not be performed because of unsuccessful catheterisation. A confident diagnosis of a stricture could not be made. Two calculi (asterisks) were noted, but exact localisation was difficult on RGU. (b) Sagittal maximum intensity projection image (multidetector computed tomographic urethrography [MDCTU]) demonstrates the membranous urethral stricture, posterior urethral calculus (short arrow), vesical calculus (arrowhead), irregular shaped bladder with contrast leakage into the perivesical tissues (double arrows) and bilateral vesico-ureteric reflux (VUR) (long arrow). (c) Sagittal multiplanar reformatting image (MDCTU) of the same patient showing part of the rectum in close proximity to the dome of the bladder, with loss of intervening fat planes and contrast noted inside the rectum (arrow), suggestive of rectovesical fistula.

Primary urethral stones may occur in the urethra proximal to an obstruction, related to chronic infective strictures and meatal stenosis in young boys.^11^ An isolated stone is often a migrated stone from the upper urinary tract.^12^ A calculus is a well-defined, sharply marginated hyperdensity within the urethral lumen. Occasionally the high density of contrast may obscure a calculus on CU unless supplemented by a control plain radiograph (Figure 11). In MDCTU, alteration of the window levels and width enables detection of the calculus within the contrast column.

### Urethral foreign bodies

**FIGURE 12:**
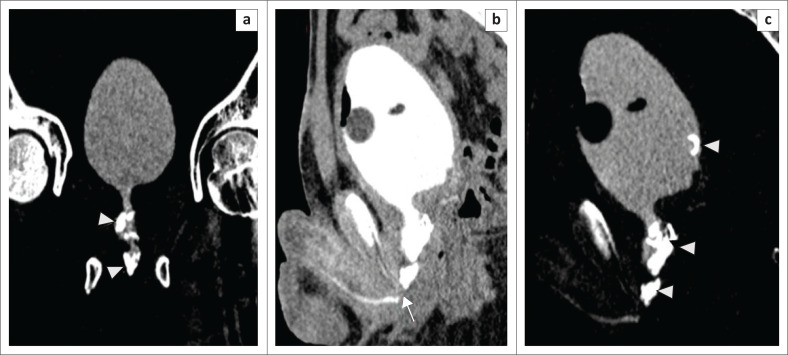
(a–c) A stricture at the bulbomembranous junction (arrow) with multiple smooth, tubular and linear hyperdensities noted in the posterior urethra (arrowheads) in all sections and also in the bladder in (c). Altering the window settings in (c), the hyperdensities not visible in (b) become markedly visible in (c). These were confirmed to be broken pieces of catheter with encrustations at surgery.

A urethral foreign body is demonstrated on MDCTU due to the inherent high-contrast resolution of CT (Figure 12).

## Bladder pathologies

### Cystitis

Bladder outlet obstruction and bladder calculus are common causes of secondary cystitis and are mostly seen as generalised and uniform bladder wall thickening. Extravasation of contrast may be identified from the inflamed bladder wall. Chronic cystitis appears as a small capacity bladder (Figure 7).

**FIGURE 13:**
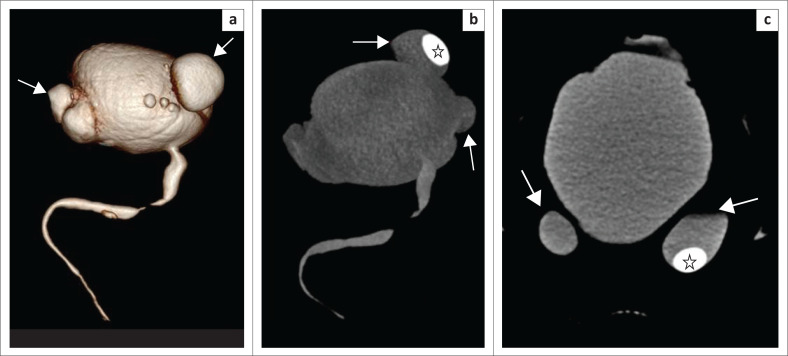
(a) Volume rendered technique, (b) maximum intensity projection and (c) axial multiplanar reformatted (MPR) images of multidetector computed tomographic urethrography. All the images are of the same patient, showing multiple bladder diverticula (arrows). A calculus (asterisks) is noted within the diverticulum on coronal and axial MPR images.

Other bladder pathologies observed are sacculations, diverticula and calculi. A focal outpouching within the bladder wall is a sacculation, and when it extends outside the wall, it is a diverticulum (Figure 13). Associated urinary stasis may lead to calculus formation within the diverticulum (Figure 13).

### Bladder rupture

**FIGURE 14:**
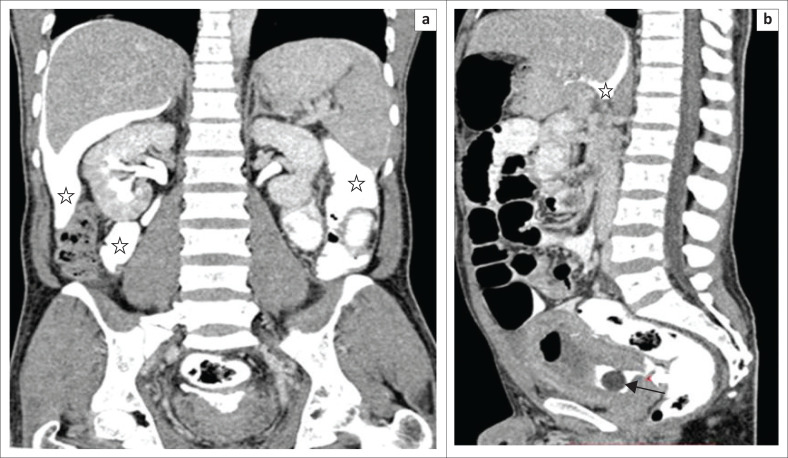
(a, b) Multidetector computed tomography coronal and sagittal reformatted images indicate rupture of the urinary bladder (arrow) with contrast material leaking into the peritoneal cavity, surrounding the bowel loops, along the paracolic gutters and reaching up to the subphrenic space (asterisks). The patient also had localised urethral injury (not shown in this image), for which he underwent MDCTU.

Bladder rupture is seen in the context of significant trauma^13,14^ and may be of two types:

- Extra-peritoneal (80% – 90%) is the result of pelvic fractures or penetrating trauma. Multidetector computed tomographic urethrography reveals a variable path of extravasated contrast material.
- Intra-peritoneal (10% – 20%) typically results from a direct blow to a distended bladder and demonstrates intra-peritoneal contrast material around the bowel loops, between mesenteric folds and in the paracolic gutters (Figure 14).

### Vesical fistula

A fistulous communication between the bladder and an adjacent structure, such as the rectum (rectovesical), colon (colovesical) or small bowel (enterovesical), is better seen on MDCTU as an irregular contrast-filled tract from the bladder to the adjacent organ. Sometimes, only a nipple-like projection outside the bladder lumen may suggest the presence of a fistulous tract (Figure 11c).

## Miscellaneous pathologies

**FIGURE 15:**
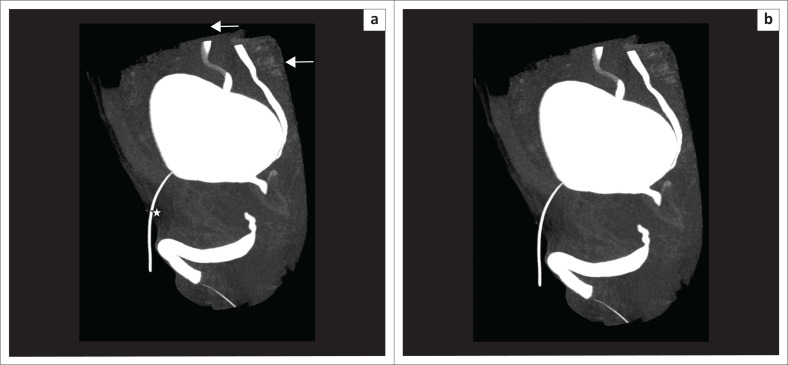
Vesico-ureteric reflux can be seen on both sides (arrows) in a patient with a posterior urethral distraction defect. Contrast in the bladder was filled through the supra-pubic catheter seen in situ (star). Retrograde filling revealed a normal anterior urethra.

**FIGURE 16:**
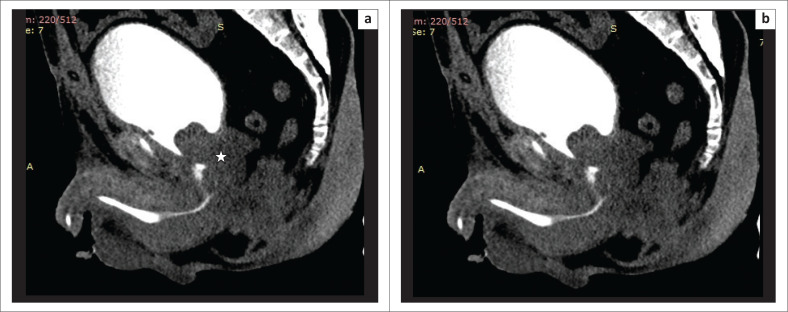
Multidetector computed tomographic urethrography: Sagittal multiplanar reformatted image showing prostatomegaly (star), with the median lobe seen bulging into the bladder base and compressing the posterior urethra, creating a false impression of a posterior urethral stricture.

Additional findings in the periurethral, vesical and pelvic soft tissues and bones may also be observed on MDCTU, including vesico-ureteric reflux (Figure 15), prostatomegaly (Figure 16), prostatic and soft-tissue calcifications, bone abnormalities and hydrocele.

## Radiation issues

The effective radiation dose on MDCTU varies between 5 millisievert (mSv) and 6 mSv,^14^ whereas in CU, it is less than 1 mSv. Radiation exposure can be further reduced on MDCTU by using a low-dose protocol, single acquisition, mA tube modulation and iterative reconstruction techniques. Although the radiation dose is higher with MDCTU, radiation exposure to the operator can be avoided, unlike in CU.

## Conclusion

This pictorial assay apprises radiologists and clinicians about the novel technique of MDCTU, its advantages over CU and appropriate patient selection to justify the increased radiation exposure, a shortcoming associated with it.

Multidetector computed tomographic urethrography is capable of comprehensive evaluation of both the anterior and posterior male urethra, periurethral tissues, urinary bladder, pelvic soft tissues and bones in a single scan, thus rendering it an effective diagnostic tool.
